# Post-traumatic Growth Dimensions Differently Mediate the Relationship Between National Identity and Interpersonal Trust Among Young Adults: A Study on COVID-19 Crisis in Italy

**DOI:** 10.3389/fpsyg.2020.576610

**Published:** 2021-01-15

**Authors:** Adriano Mauro Ellena, Giovanni Aresi, Elena Marta, Maura Pozzi

**Affiliations:** Department of Psychology, Catholic University of the Sacred Heart, Milan, Italy

**Keywords:** COVID-19, collective trauma, social identity, national identity, post-traumatic growth, interpersonal relationships

## Abstract

**Background:**

In Italy, the COVID-19 pandemic has caused a collective trauma. Post-traumatic growth (PTG) has been defined as the subjective experience of positive psychological changes as a result of a traumatic event. PTG can involve changes in five psychological main dimensions: relating to others, new possibilities, personal strength, spiritual change, and appreciation of life. In the context of national emergencies, those PTG dimensions encompassing changes at the social level (e.g., relating to others) can play a role in coping strategies that involve a renewed sense of self and one’s social identities, including national identities, and in turn, foster a stronger sense of trust and connection to others.

**Aim:**

To investigate how each of the five PTG dimensions mediates the association between the salience of national identity and interpersonal trust in a sample of Italian young adults. Trust in national and European institutions were expected to positively predict the strength of the Italian national identity which in turn was expected to be positively associated with interpersonal trust, and the PTG relating to others dimension to mediate this association.

**Method:**

This study involves the secondary analysis of data from a representative sample of 2,000 Italian young adults (age range 18–34 years). Participants completed a web survey during the peak of the COVID-19 crisis in Italy with measures of trust in EU and national institutions, national identity, interpersonal trust, and the PTG Inventory. Structural equation modeling procedures were employed for key hypotheses tests.

**Results:**

Trust in national institutions positively predicted national identity, which in turn was positively associated with interpersonal trust. Evidence of a full mediation effect of the PTG relating to others dimension on the association between national identity and interpersonal trust was found.

**Discussion:**

Findings contribute to clarify the psychological responses to collective traumas. In the context of Italy’s COVID-19 crisis, trust in national institutions reinforced Italian national identity, which was in turn associated with greater interpersonal trust, but only when psychological responses to the trauma involved changes in how individuals perceived and related to others, and not merely a focus on the self. Theoretical and practical implications are discussed.

## Introduction

Italy was among the most heavily affected countries by the COVID-19 pandemic. Since February 21, 2020, when the first case of the virus contracted by a person not coming from China was registered, the government has taken a series of increasingly restrictive measures to contain the spread of the virus. At first, restrictions were limited to specific areas but were soon extended to the whole country on March 8 when a lockdown was imposed at the national level (Governo Italiano, 2020). It was mandatory to stay at home, unless there were proven reasons of necessity. Italy was the first European country to issue such a nationwide stay-at-home order.

As of April 30, 2020, 101,551 positive cases were documented out of the 205,463 people tested positive from the beginning including 19,843 admitted to the hospital and 1,694 of which were receiving intensive care, and the death toll reached 27,967. In the provinces most affected by the pandemic, such as Bergamo and Brescia, March and April monthly mortality rates raised up to 500% as compared with the previous year. The whole country was shocked by the images from Bergamo where army trucks were moving coffins of COVID-19 victims to other regions who could no longer be accommodated into local cemeteries. All this happened in a context of isolation, fear of an unknown invisible entity, and uncertainty toward the future. At the same time, media exposure of the pandemic was overwhelmingly contributing to an increase of anxiety and depression in the population with potential traumatic effects for many (Garfin et al., 2020). At the time of writing this paper, many European countries, including Italy, are going through a second wave that reached its peak around the end of November. As of November 30, 2020, Italy has lost more than 55,000 people and 33,000 people are still hospitalized, of which 3,700 are in intensive care. The national emergency is still ongoing and lockdown measures are in place again after a relaxation in the summer.

Despite the fact that traumas are usually individual experiences, there are circumstances in which they are experienced by entire communities bringing out collective traumatic dynamics (Alexander et al., 2004). Circumstances generally include natural disasters, such as hurricanes (Lowe et al., 2013; Manove et al., 2019), earthquakes (Wlodarczyk et al., 2016), and, for what concerns this work, epidemics. Given its health and social impact on the Italian population, the COVID-19 pandemic presents characteristics of a collective trauma experience. According to Kinston and Rosser (1974), this is a situation of massive collective stress. Others (Cuzzolaro and Frighi, 1998) defined an event to be traumatic when it impacts a community overall causing social, emotional, and behavioral reactions. Crocq (1987) emphasized the characteristics of brutality and immediacy. More recently, Hirschberger (2018) highlighted the crisis of meaning that follows collective traumas. According to the author, as individual trauma changes the view of oneself and the perception of the world, collective traumas disrupt social contexts as well as intra-group and inter-group relations. Thus, the sense and meaning that people attribute to their world can be dramatically changed (Updegraff et al., 2008).

By examining the COVID-19 emergency in Italy, this paper will reflect on the psychological implications of collective trauma, both in terms of the effects of individuals’ social identity at the national level and the interplay with different dimensions of personal growth in response to such trauma.

### Collective Trauma, National Identity, and Trust Toward Others

During collective traumatic experiences, community members are exposed to events that leave indelible signs in their group consciousness through the construction of new collective memories (Alexander et al., 2004). According to Tajfel and Turner (1979), these new collective memories facilitate both the formation of new social identities and the strengthening and salience of pre-existing ones. There is evidence that strong and salient identities contribute to effective coping with stressful events (Kira et al., 2019) and help in safeguarding mental health (Berzonsky, 1992) because they mediate the relationship between perceived threat and psychological well-being (Schmid and Muldoon, 2015). In other words, when their social group is threatened, people tend to strengthen their social identities which in turn play a significant role in coping processes to traumatic events and dealing with the adverse event and anxiety that results from it (Griswold et al., 2018).

National identity is the type of social identity that derives from the sense of belonging to a nation or a state (Triandafyllidou, 1998). The construction of national identity is a complex phenomenon in which many aspects of a subject’s social life interact. It evolves gradually during infancy and adolescence to become an integral part of our self. National identity originates from categorization processes and allows people to differentiate between different groups of nations. The division of the world into categories permits people to distinguish between what is considered home and what is foreign, thus acquiring territorial and cultural values. National identities derive from a perception of belonging which implies cognitive, evaluative, and emotional processes, both at the individual and social level. This allows people to identify with their significant national traits, thus regulating their behavior. Through national identity, people share beliefs, representations, and values that contribute to foster emotional bonds with compatriots (Smith, 1992).

It is important to stress that national identities may have practical implications. The consequences of the categorization process, such as the outgroup homogeneity effect and the ingroup bias, also materialize in the distinctions between the different national groups (Tajfel and Turner, 1986): one’s own nation will be perceived as more varied in comparison with foreign nations, which will instead be more likely to be stereotyped. Nationalism, for example, often derives from the intergroup confrontation and breeds from favoritism toward the ingroup. Radical forms of nationalism include elevating one’s nation above the others, judging it as superior, and legitimizing its dominance over the other groups. This may have consequences including discriminant behaviors and rejection toward members of other national and ethnic groups (e.g., the immigration issue in Europe) (Smith, 1992). At the same time, national identity can enhance closeness among people which in turn boosts social support. People’s national identity can become salient in cases whereas during the COVID-19 pandemic, the entire nation is affected by a traumatic event involving its citizens as a whole. Facing a common internal or external, natural or biological threat is supposed to enhance people’s feelings of being part of a nation (West and Smith, 1997; Guibernau, 2004). As previously stated, this national sentiment became evident during collective manifestations such as singing in the balconies and support networks’ activation (e.g., volunteering, elderly care, psychological support), and contributed to people coping with the negative emotions that the situation triggered (Venuleo et al., 2020).

The idea of nation can be identified with its institutions resulting in the establishment of in-group boundaries (Jelen, 2011), and fostering a sense of belonging and national identity (Harttgen and Opfinger, 2014). According to Gibson and Condor (2009), national institutions can be considered as hybrid entities including group of peoples, material objects (buildings, places), and procedures (laws, constitutions, government measures). In times of emergency and threat, institutional representatives often emphasize the notion of “we” as a nation with the intent of strengthening people’s national identity. Moreover, if people trust the government and its institutions, the sense of belonging to the most salient social group at that time, the national one, is generally stronger. In Italy, the pandemic has been managed for the most part by national institutions such as the government, the National Health System (NHS), the police, and the civil protection agency. The European Union (EU) had a relatively marginal role and it has often been considered as an entity external to the nation.

The implications of social identities in regards to relations with others have been extensively examined (Tyler, 2001; Tanis and Postmes, 2005; Voci, 2006). It has been widely acknowledged that threatening situations, such as pandemics, strengthen the salience of social identities and foster solidarity, cooperation, and norm compliance within the group (Dovidio et al., 2020). For what concerns national identity, it has been demonstrated that its salience and strength are positively related to trust toward other people and cooperative behavior, and this is especially true under conditions of risk and uncertainty (Brewer, 2008; Ntontis and Rocha, 2020). Interpersonal trust can be considered as a positive expectation toward others’ behavior (Robinson, 1996) and the sincerity of their words (Hosmer, 1995). Trust is important both during an emergency and the following period of recovery because it helps people to coexist and cooperate (Righetti and Finkenauer, 2011). In fact, as Kramer and Wei (1999) highlighted, in conditions of social identity threat, interpersonal trust is based more on common membership of the salient group (identification-based trust) rather than on individual reciprocal benefits (calculus-based trust), thus explaining the link between one’s social identities and how we relate with others.

### Post-traumatic Growth

Because of its fundamental importance in mental health, trauma has been extensively analyzed in the psychology literature (Prati and Pietrantoni, 2006; Luszczynska et al., 2009). Despite the majority of studies focused on the psychopathological consequences of traumatic events at the individual level, others have examined positive psychological and social changes after trauma and adverse experiences (Linley and Joseph, 2004). Post-traumatic growth (PTG) (Tedeschi et al., 1998) has been defined as the subjective experience of positive psychological changes in the aftermath of a traumatic experience (Tedeschi and Calhoun, 1995). Importantly, PTG is not a monolithic construct, but it encompasses five distinct dimensions (Prati and Pietrantoni, 2009): changes in how people relate with others (relating to others: i.e., an increased willing to express emotions or even accepting more likely help from others), recognition of new possibilities (new possibilities: i.e., seen as an increased attitude to take new paths in life and redefine priorities), a sense of greater personal strength (personal strength: i.e., improved sense of self-efficacy, strength, and self-confidence), changes toward spirituality (spiritual change: i.e., religious beliefs, spiritual matters, and existential/philosophical questions), and greater appreciation of life (appreciation of life: i.e., considering meaningful and worth in life’s little things).

Some studies (Wasco et al., 2002; Cacioppo et al., 2015) highlighted how a collective experience of trauma can help people reflect on their traumatic experiences and consequently learn from them. Social identities are involved in this process (Calhoun and Tedeschi, 2012; Tedeschi et al., 2018; Muldoon et al., 2019): a renewed sense of belonging to a group or community, including the national level, significantly contributes to the meaning-making process. In turn, it is crucial for the positive processing of trauma and its implications in terms of social interactions, which include increased self-disclosure and trust within social relationships.

In summary, it has been established that social identities, including the national level, have implications in regard to how individuals relate to others and put their trust into them. What is lacking is a deeper understanding of the interplay between social identities and PTG in determining such coping responses to emergency situations that involve the social sphere. Importantly, despite its multidimensional nature that encompasses both individual and social aspects, the PTG has generally been examined in the literature as a unique dimension. This may have resulted in overlooking the role of each specific dimension. Those dimensions encompassing changes at the social level (i.e., the relating to others dimension) may play a role in the connection between social identities and responses of trust toward others, whereas the other dimensions involving changes at the individual level (e.g., personal strength) may not.

### Aims

**FIGURE 1:**
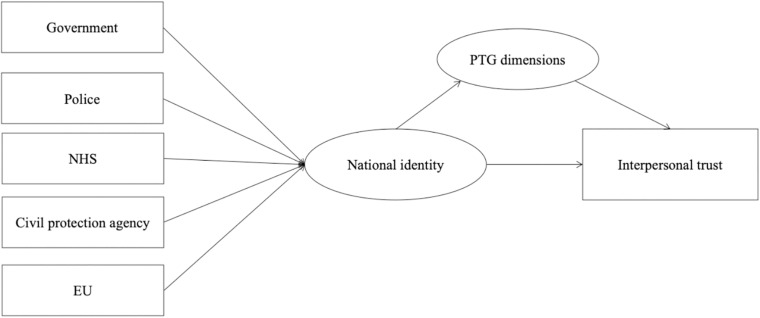
Conceptual model.

This study investigated how each of the five post-traumatic growth dimensions mediates the association between the salience of national identity and interpersonal trust in a sample of Italian young adults. Trust in national and European institutions were expected to positively predict the strength of the Italian national identity (Hypothesis 1), which in turn was expected to be positively associated with interpersonal trust, and the PTG relating to others dimension to mediate this association (Hypothesis 2). Because the other PTG dimensions involve changes at the individual level (i.e., personal strength and appreciation of life, new possibilities, and spiritual change), they were expected to play a less significant role (Hypothesis 3). Figure 1 depicts the overall conceptual model.

## Materials and Methods

### Data

This study involves the secondary analysis of data collected by the Osservatorio Giovani of the Istituto Toniolo di Studi Superiori (Milan, Italy). Since 2012, the Osservatorio conducts yearly computer-assisted web interview (CAWI) surveys regarding topics related to young people, such as social and economic inclusion of people that are Not in Employment, Education, or Training (NEET), as well as healthy behaviors, cultural issues, and participation (Alfieri et al., 2015; Marta et al., 2016; Aresi et al., 2018, 2020; Pistoni et al., 2019). The authors contributed to the design of the major study. Sampling and data collection were conducted by Ipsos s.r.l. between March 27 and March 31, 2020 during the peak of the COVID-19 crisis in Italy. Out of 4,116 contacts, a sample of 2,000 responders was achieved to be representative of the Italian young adult (18 to 34 years old) population in respect of several sociodemographic characteristics including gender, age range, educational level, occupation status, and geographic area (Istituto Nazionale di Statistica, 2020). About half (49%) of the respondents were female and mean age was 27.1 (SD = 4.69). The sample consisted of the following age ranges: 18–22 (19.3%, *N* = 387), 23–25 (18.9%, *N* = 379), 26–28 (19.9%, *N* = 398), 29–31 (19.9%, *N* = 398), and 32–34 (21.9%, *N* = 438). Participants’ occupation status was distributed as follows: 54.3% workers, 36.9% students, and 8.9% unemployed. In terms of geographic area, 24.8% lived in the Northwestern, 17.8% in the Northeastern, 19.2% in the Central, and 38.2% in the Southern region.

### Measures

#### Trust in National Institutions

Trust in institutions was measured by asking: “Since the beginning of COVID-19 emergency how has your confidence in these institutions changed?” A 5-point Likert scale was used with values ranging from 1 (“Strongly increased”) to 5 (“Strongly decreased”). Scores were reversed before data analysis. The following institutions were considered: the government, the NHS, the police, the civil protection agency, and the EU.

#### National Identity

To assess Italian national identity, the In-Group Identification Scale was adapted (La Barbera and Capone, 2016). Specifically, the sub-scales Satisfaction (four items), Solidarity (three items), and Centrality (three items) were used. Items were rated using a 10-point Likert scale with values ranging from 1 (“Totally disagree”) to 10 (“Totally agree”). Examples of items are as follows: “I’m glad to be Italian” (Satisfaction); “I feel committed to Italians” (Solidarity); “The fact that I’m Italian is an important part of my identity” (Centrality). All three subscales were of high reliability: Satisfaction (α = 0.81); Solidarity (α = 0.79); Centrality (α = 0.79).

#### Post-traumatic Growth

To measure post-traumatic growth, the Italian version of the PTG Inventory (PTGI) was used (PTGI; Prati and Pietrantoni, 2014). The scale has demonstrated construct validity. It consists of five subscales measuring perceptions of trauma-induced changes in (1) relating to others (example of item: “I learned a great deal about how wonderful people are”), (2) new possibilities (example of item: “I developed new interests”), (3) personal strength (example of item: “I have a greater feeling of self-reliance”), (4) spiritual change (example of item: “I have a better understanding of spiritual matters”), and (5) appreciation of life (example of item: “I have a greater appreciation for the value of my own life”). Respondents were prompted by asking “Compared to before the COVID-19 emergency, how has your position changed today with respect to the following statements?” Items were rated using a 5-point Likert scale with values ranging from 1 (“Much less than before”) to 5 (“Much more than before”), and 3 considered the midpoint (“No changes”). In the validation study, the PTGI showed acceptable reliability for both the total score (α = 0.93) and the subscales, with Cronbach’s alphas for the subscales relating to others (α = 0.86), new possibilities (α = 0.84), personal strength (α = 0.80), spiritual change (α = 0.78), and appreciation of life (α = 0.74).

#### Interpersonal Trust

A single item adapted from Zmerli and Newton (2008) was used to measure levels of interpersonal trust: “Since the start of COVID-19 emergency, how has your attitude toward this statement changed? Most people are trustworthy”. A 5-point Likert scale was used with values ranging from 1 (“Strongly increased”) to 5 (“Strongly decreased”). Scores were reversed before data analysis.

### Data Analysis

Descriptive statistics (mean and proportions) were calculated, and the data were checked for normality. We analyzed the construct validity (i.e., measurement model) of the Italian national identity and the five PTG dimensions through confirmatory factor analysis. In accordance with the hypothesized conceptual model, we performed structural equation modeling to investigate predictors of national identity and the relationship between the latter and interpersonal trust. The mediating effect of each of the five PTG dimensions was tested using the accelerated-bias-corrected bootstrap estimation procedure, which yields the most accurate confidence intervals (CIs) for the indirect effects (MacKinnon et al., 2004). In the procedure, the given sample size was randomly resampled 10,000 times with replacement, and then 10,000 estimations of the indirect effect were calculated. When the 95% CI for an indirect effect did not include zero, the indirect effect was significant. The overall fit of the model was evaluated considering the values for acceptable absolute, relative, and parsimony fit indices. Selection of these indices was based on their statistical power and widespread use in relevant statistical literature (Hu and Bentler, 1999; Ullman, 2006; Kline, 2011). As indicative of absolute fit, we considered the root mean square error of approximation (RMSEA < 0.08) and the standardized root mean square residual (SRMR < 0.08). As a relative fit index, we used the values of the comparative fit index (CFI > 0.90) and the Tucker–Lewis index (TLI > 0.90) (Hu and Bentler, 1999; Ullman, 2006; Kline, 2011). Mplus version 7 (Muthén and Muthén, 1998-2015) was used to analyze data.

## Results

### Descriptive Statistics and Measurement Model

Table 1 displays the means, SDs and correlations among measured variables. In our sample, we found medium to high levels of national identity. This is comparable with those found by La Barbera and Capone (2016). In regard to PTG dimensions, we found that scores of “appreciation of life” were the greatest and of “spiritual change” the lowest. The result on the latter dimension is consistent with the validation study, although Prati and Pietrantoni (2014) found that the “relating to others” dimension scored the highest.

**TABLE 1:** Descriptive statistics and correlations between measured variables.

Variable	*M*	SD	1	2	3	4	5	6	7
1. National identity	6.40	2.01	–						
2. Interpersonal trust	2.76	0.89	0.171***	–					
3. PTG relating to others	3.23	0.56	0.326***	0.238***	–				
4. PTG new possibilities	3.30	0.56	0.247***	0.129***	0.634***	–			
5. PTG personal strength	3.31	0.61	0.231***	0.075**	0.558***	0.681***	–		
6. PTG spiritual change	2.97	0.76	0.181***	0.124***	0.409***	0.282***	0.227***	–	
7. PTG appreciation of life	3.47	0.65	0.280***	0.073*	0.559***	0.648***	0.594***	0.238***	–

*N* = 2,000, *p > 0.05, **p > 0.01, ***p > 0.001.

The data showed a normal univariate distribution, given that most skewness values and kurtosis values fell within the range of −1.0 to +1.0. As hypothesized, national identity scores were positively related to interpersonal trust and the five PTG dimensions. In addition, each PTG dimension was positively associated with interpersonal trust.

The measurement model for the six latent variables (national identity and the five PTG dimensions) was tested, and the results revealed an acceptable fit to the data: [*χ*^2^(419) = 2,111.028, *p* < 0.001, CFI = 0.948, TLI = 0.942, RMSEA = 0.045, SRMR = 0.031]. All the standardized factor loadings for the indicators on the latent variables were statistically significant (|λ| ranging from 0.55 to 0.87, *ps* < 0.001), signifying that the six latent variables were well represented by their respective indicators. Given the adequacy of the measurement model, the structural model was examined next.

### Structural Model and Mediation Analyses

**FIGURE 2:**
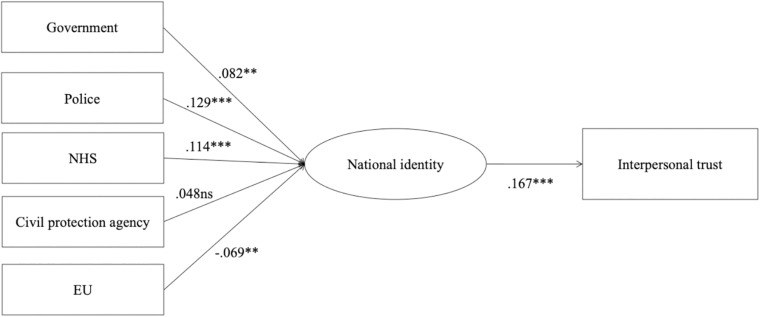
Structural model. Measurement model not displayed for parsimony. Standardized coefficients are reported. ^∗∗^*p* < 0.01, ^∗∗∗^*p* < 0.001, ns, non significant.

The structural model was estimated modeling trust in the EU and the four national institutions as predictors of national identity, and national identity as a predictor of interpersonal trust as the outcome variable. The indices in the estimated model revealed an acceptable fit for the observed data (CFI = 0.944; TLI = 0.934; RMSEA = 0.075; SRMS = 0.029). As presented in Figure 2, trust in national institutions, except the civil protection agency, positively predicted national identity. Conversely, trust in EU institutions negatively predicted national identity. Thus, Hypothesis 1 was partially confirmed. The effect of national identity on interpersonal trust was positive and significant (*β* = 0.167, *p* < 0.001).

**FIGURE 3:**
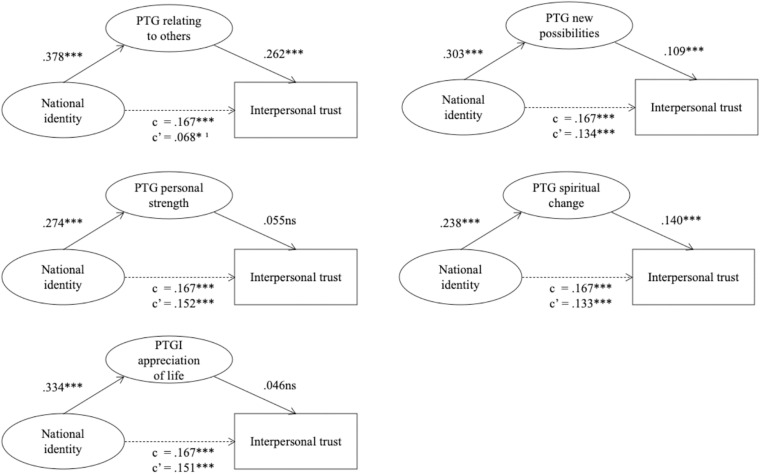
Mediation models. Measurement model not displayed for parsimony. Standardized coefficients are reported. c, total effect; c’, direct effect. ^∗^*p* < 0.05, ^∗∗∗^*p* < 0.001; ns, non significant; ^1^95% CIs include zero.

We conducted five separate models to examine whether each of the five PTG dimensions would serve singly as a mediator in the relationship between national identity and interpersonal trust (Figure 3). All models evidenced an acceptable fit to the data. The results showed that the PTG relating to others, perceptions of having new possibilities, and spiritual change dimensions mediated the positive relationship between national identity and interpersonal trust, whereas personal strength and appreciation of life did not (both indirect effects were not significant). The indirect effect of the relating with others dimension on interpersonal trust was significant [indirect effect = 0.99, *p* < 0.001, 99% CI = (0.061, 0.136)], as well as that of perceptions of having new possibilities [indirect effect = 0.33, *p* < 0.01, 99% CI = (0.005, 0.061)] and spiritual change [indirect effect = 0.33, *p* < 0.01, 99% CI = (0.08, 0.058)]. In regard to the relating with others dimension, inspection of CIs suggests that, despite the direct effect of national identity on interpersonal trust remained significant at a 0.05 *p*-value [*β* = 0.068, *p* < 0.05, 99% CI = (−0.002, 0.138)], it was markedly decreased and CIs overlapped zero. Therefore, this mediation can be considered full (Hypothesis 2). Conversely, after accounting for the mediating effect of perceptions of having new possibilities and spiritual change dimensions, the direct effect was only slightly decreased and CIs did not include zero [*β* = 0.134, *p* < 0.001, 99% CI = (0.065, 0.202), *β* = 0.133, *p* < 0.001, 99% CI = (0.067, 0.200), respectively]. Thus, these are partial mediations (Hypothesis 3).

## Discussion

This work has examined some implications of the COVID-19 pandemic in Italy from a social identity perspective. Given the collective nature of this public health emergency, national identity was expected to be salient and to be involved in coping processes that included cooperative behavior and trust toward other people (Griswold et al., 2018; Kira et al., 2019; Dovidio et al., 2020). During the first weeks of the health crisis, feelings of national identity were manifested through people hanging national flags on balconies and performing traditional Italian music out of their windows with neighbors. These expressions can be considered a way of reacting to the anxiety and fear that the epidemic was evoking, thus highlighting the coping strategy feature of the shared social identity (Haslam et al., 2014). The more people feel part of a group, the more capable they feel of facing adversities (Scarf et al., 2016).

First, our study indicates which aspects of their life and personal growth people gave the most thoughtful attention to during the first phase of the health emergency. The “appreciation of life” dimension received the highest score and “spiritual change” the lowest. We speculate that this is because the virus represented a threat to people’s health and was potentially fatal, thus accentuating concerns about one’s life and physical integrity. Spirituality was involved to a lower extent. The literature presents mixed findings on this matter. On the one hand, because the COVID-19 pandemic significantly altered the standard way of life, people may have been more prone to reflect on spiritual matters, thus allowing spirituality to be a resource to cope with stress (del Castillo, 2020). On the other hand, according to a new Pew Research Center survey (Gecewicz, 2020), a minority of people have changed their spirituality or religious behavior during the health emergency so far. We can hypothesize that for young people to reflect on spiritual issues, such as life’s meaning, requires more complex processes and more time than an immediate greater “appreciation of life” would require. Longitudinal data could offer insight on whether, after the turmoil of the pandemic’s first phase, young people have reflected on their spirituality to a greater extent. Interestingly and contrary to previous studies (Prati and Pietrantoni, 2014), we found the “relating to others” dimension scored the lowest. This result is likely to be due to the substantial reduction of social interactions that people experienced during the lockdown.

For what concern national identity, our results confirm the importance of the national identity by the clear distinction between feelings toward national and supranational (i.e., the EU) institutions. The greater the trust in the Italian government and national institutions highly involved in the management of the crisis was, the feelings of national identity increased. However, the opposite was found for the EU suggesting that trust in this supranational institution clashed with feelings of “we” as a nation. In light of Italians’ ambivalent feelings toward the EU, this institution was perceived as an external entity that did little to support Italy during the emergency and was therefore considered an outgroup by most Italians (Laflan, 2004). This was likely to be further accentuated by the fact that during the first weeks of the pandemic, the media in other European countries portrayed the Italian government and national health system as culpably unprepared to cope with the virus, thus strengthening in-group boundaries increasing the gap between an “us” (ingroup) and a “them” (outgroup) (Jelen, 2011). In other words, these conjoint factors strengthened the salience of a social identity as Italians and in its national institutions in contrast to supranational ones (Ruiz Jiménez et al., 2004; Harttgen and Opfinger, 2014). To promote national identity as a resource capable of conveying feelings of belonging, union, closeness, and solidarity, institutions could communicate an all-encompassing and supportive idea of “us,” and avoid evoking feelings of hate, discrimination, and fear for the “other.” As such, national identity would become an asset and not an obstacle for cooperation, allowing people to manage fear and anxiety by building networks, including virtual ones, and participating in collective events based on positive values. The result would likely be greater reflexivity on the health emergency and its social and psychological consequences, possibly accompanied by greater psychological growth (Venuleo et al., 2020).

By indicating that social identity salience can lead to a more generalized trust in interpersonal relationships in an emergency context, our findings are consistent with those of previous studies on this issue (Brewer, 2008; Ntontis and Rocha, 2020). Their novelty lies in the nuanced examination of the mediation role in this association played by each of the five post-traumatic growth dimensions.

Four out of the five PTG dimensions appear to be at best weakly involved, and the most “social” dimension (i.e., relating to others) only fully mediated the national identity—interpersonal trust association. This means that without a re-attribution of meaning to one’s relationships with others, renewed social identities do not result in positive changes in how people relate with others. In other words, trust in interpersonal relationships does not come automatically from salient social identities, but is the outcome of a meaning-making process whereby people go through a process of reflection and growth, especially when it comes to relationships with others (Muldoon et al., 2019). Furthermore, because collective traumas affect social bonds, during emergencies where other people can be considered a threat (in this specific case: a source of contagion), understanding that they can be part of the solution and not part of the problem is fundamental to cope with it.

In regard to the other PTG dimensions, a renewed spirituality and the perception of having new possibilities in life demonstrated to have a partial and modest role in mediating the association between national identity and trust toward others. We speculate that changes in these dimensions may involve the way people see others and the relationships they establish with them, i.e., seeing others as brothers and sisters in a religious community, thus explaining this effect of partial mediation (Tedeschi et al., 2018). Both PTG dimensions of personal strength and appreciation of life include changes closely related to the individuality of each person, i.e., the perception of being stronger and able to better accept negative things, as well as the value that people give to their lives and the ability to appreciate the positives of every day (Tedeschi et al., 1998). Despite these positive outcomes of the process of personal growth after a traumatic experience, we have demonstrated that they do not play a significant role in reshaping how people see others and relate with them.

### Limitations

The present study contains some limitations that are noteworthy, the most important being its cross-sectional nature. Further research with longitudinal designs is needed to better examine the causal relationships and mediation effects. Besides, this study did not consider the variables that can promote post-traumatic growth which are necessary to understand better the design of clinical and community interventions that create psychological well-being. Lastly, data of this study came from a sample of Italian young adults and it is unclear to what extent results encompass other age cohorts and countries in Europe.

### Conclusion

Findings of this study contribute to the literature by providing empirical evidence to the notion that, in a collective emergency and trauma context, the salience of national social identities is related to a more generalized trust in interpersonal relationships. This can also be understood from the perspective of living a “common fate” in the face of mortal danger and can determine a growth in a more relational and prosocial dimension, which in turn impacts on general trust toward others and consequently on well-being (Haslam et al., 2014).

Results of this study have implications for the work of social and clinical psychologists. Reinforcing positive identities through psychosocial interventions and consultation can be the basis for psychological growth at an individual and community level (Haslam et al., 2008; Yakushko et al., 2008; Alexander Haslam, 2014). This is because salient and positive social identities offer greater access to solidarity and social support (Muldoon et al., 2019), and play a protective role toward stressors resulting in more favorable psychological outcomes (Zoellner and Maercker, 2006). Those who have experienced trauma affirm that they need to express their feelings and confide their emotions to someone (Lehman et al., 1986), and there is evidence that sharing thoughts about traumatic experiences with others contribute to a better post-traumatic adjustment and more favorable outcomes (Pennebaker, 1989, 1993). Moreover, having valid social support appears to be a predictor of post-traumatic growth which in turn represents a healthy way to respond to trauma (Nolen-Hoeksema and Davis, 1999; Lev-Wiesel et al., 2004; Ramos and Leal, 2013). On this basis, it seems crucial to raise clinician awareness’ of the possibility of growth to encourage the development of those elements that predict the outcome in clinical practice.

## Data Availability Statement

The data analyzed in this study is subject to the following licenses/restrictions: The data underlying this article were provided by Osservatorio Giovani© of the Istituto Toniolo di Studi Superiori©, and will be shared on request to the corresponding author with permission of the Osservatorio. Requests to access these datasets should be directed to corresponding author.

## Ethics Statement

The studies involving human participants were reviewed and approved by the Osservatorio Giovani© of the Istituto Toniolo di Studi Superiori©. The patients/participants provided their written informed consent to participate in this study.

## Author Contributions

AE, MP, and EM designed the study. GA analyzed the data. AE and GA wrote the manuscript. MP and EM contributed to revisions. All authors read and approved the final manuscript.

## Conflict of Interest

The authors declare that the research was conducted in the absence of any commercial or financial relationships that could be construed as a potential conflict of interest.
